# Evaluating Inpatient Treatment Outcomes of Eating Disorders at Ty Llidiard General Adolescent Unit: Service Evaluation Project in South Wales

**DOI:** 10.1192/bjo.2025.10502

**Published:** 2025-06-20

**Authors:** Mohamed Marey, Ceri Price

**Affiliations:** Cwm Taf Morgannwg University Health Board, Rhondda Cynon Taf, United Kingdom

## Abstract

**Aims:** Adolescents with eating disorders often require intensive inpatient care, and pharmacotherapy, including olanzapine, has been proposed as an adjunct to support weight restoration and reduce psychological symptoms such as food-related anxiety. However, evidence on olanzapine’s effectiveness in real-world adolescent settings remains limited. Ty Llidiard is the only inpatient general adolescent unit covering the whole of South Wales. The aim of this project is to evaluate whether Ty Llidiard provides effective medical care for its patients with eating disorders. The primary aim is evaluating weight restoration achieved as well as overall improvement in functioning. The secondary aim is evaluating whether the use of olanzapine is effective in achieving the primary aim.

**Methods:** A retrospective evaluation was conducted on all adolescents admitted to Ty Llidiard unit between May 2018 and December 2023 with a primary diagnosis of an eating disorder. Data collected included demographic information, length of stay, changes in %mBMI, and functional outcomes as measured by the Children’s Global Assessment Scale (CGAS). Comparisons were made between patients receiving olanzapine and those managed without pharmacotherapy. Data anonymization protocols ensured confidentiality. All patients admitted to the unit over the study period were included which eliminates selection bias.

**Results:** The cohort comprised 93 patients. The average length of stay was 105 days, during which patients achieved a mean weekly weight gain of 1% mBMI (approximately 0.5 kg per week) and an overall increase in %mBMI of 13.1%. Functional improvements were observed, with CGAS scores increasing from admission to discharge with an overall increase of 18.9 points. However, no significant differences in weight restoration or CGAS improvements were found between the olanzapine and non-pharmacotherapy groups. This result was no different when the analysis was limited to detained patients (marker of severity) or included those with any diagnosis of ED (not just as a primary diagnosis). There was more use of MHA in more unwell patients.

**Conclusion:** Ty Llidiard unit demonstrated effective treatment for weight restoration and functional improvement in adolescents with eating disorders. Evidence from RCTs and meta-analyses on olanzapine use in adolescents with eating disorders presents mixed findings. However, the findings from Ty Llidiard indicate that olanzapine does not provide additional benefits for these outcomes. These results challenge the routine use of pharmacotherapy in high-acuity inpatient settings and underscore the need for further research into the role of olanzapine in adolescent eating disorder treatment. This provides real-world generalisable information, especially for clinicians working in specialist inpatient services.

